# Emerging Technologies for Degradation of Dichlorvos: A Review

**DOI:** 10.3390/ijerph18115789

**Published:** 2021-05-28

**Authors:** Yuming Zhang, Wenping Zhang, Jiayi Li, Shimei Pang, Sandhya Mishra, Pankaj Bhatt, Daxing Zeng, Shaohua Chen

**Affiliations:** 1 State Key Laboratory for Conservation and Utilization of Subtropical Agro-Bioresources, Guangdong Province Key Laboratory of Microbial Signals and Disease Control, Integrative Microbiology Research Centre, South China Agricultural University, Guangzhou 510642, China; 20193138058@stu.scau.edu.cn (Y.Z.); 20191047008@stu.scau.edu.cn (W.Z.); jiayilee@stu.scau.edu.cn (J.L.); 20192047012@stu.scau.edu.cn (S.P.); sandhyamanshi@gmail.com (S.M.); pankajbhatt.bhatt472@gmail.com (P.B.); 2 Guangdong Laboratory for Lingnan Modern Agriculture, Guangzhou 510642, China; 3 School of Applied Chemistry and Biological Technology, Shenzhen Polytechnic, Shenzhen 518055, China

**Keywords:** dichlorvos, biodegradation, degradation pathways, mechanisms

## Abstract

Dichlorvos (*O*,*O*-dimethyl *O*-(2,2-dichlorovinyl)phosphate, DDVP) is a widely acknowledged broad-spectrum organophosphorus insecticide and acaracide. This pesticide has been used for more than four decades and is still in strong demand in many developing countries. Extensive application of DDVP in agriculture has caused severe hazardous impacts on living systems. The International Agency for Research on Cancer of the World Health Organization considered DDVP among the list of 2B carcinogens, which means a certain extent of cancer risk. Hence, removing DDVP from the environment has attracted worldwide attention. Many studies have tested the removal of DDVP using different kinds of physicochemical methods including gas phase surface discharge plasma, physical adsorption, hydrodynamic cavitation, and nanoparticles. Compared to physicochemical methods, microbial degradation is regarded as an environmentally friendly approach to solve several environmental issues caused by pesticides. Till now, several DDVP-degrading microbes have been isolated and reported, including but not limited to *Cunninghamella*, *Fusarium*, *Talaromyces*, *Aspergillus*, *Penicillium*, *Ochrobium*, *Pseudomonas*, *Bacillus*, and *Trichoderma*. Moreover, the possible degradation pathways of DDVP and the transformation of several metabolites have been fully explored. In addition, there are a few studies on DDVP-degrading enzymes and the corresponding genes in microorganisms. However, further research relevant to molecular biology and genetics are still needed to explore the bioremediation of DDVP. This review summarizes the latest development in DDVP degradation and provides reasonable and scientific advice for pesticide removal in contaminated environments.

## 1. Introduction

The overly common use of organophosphorus pesticides (OPs) has led to a high risk of exposure to acute toxic compounds for various kinds of creatures, including humans [1]. As a representative organophosphorus pesticide, dichlorvos (*O*,*O*-dimethyl *O*-(2,2-dichlorovinyl)phosphate, DDVP) has been commonly used in developing countries and many other regions for more than 40 years [2]. DDVP has the molecular formula C_4_H_7_Cl_2_O_4_P, with a molecular weight of 220.98, vapor pressure of 1.2 × 10^–2^ mmHg at 20 °C, and density of 1.415 g/mL at 25 °C. It is classified by the World Health Organization (WHO) as class 2B: possible carcinogens [3]. In addition, the United States Environmental Protection Agency (EPA) has also classified it as a Class Ⅰ pollutant (highly toxic) [4].

Since DDVP came into commercial use in 1961, it could be seen in many countries due to its significant advantages in terms of controlling internal and external parasites in crops and livestock and its ability to eliminate several pests in houses and farmlands [1]. The yearly sales in 2019 around the world were about USD 88 million [5]. In many developing countries, the excessive use or misuse of DDVP in agricultural production leads to serious environmental problems and hazardous conditions. This situation usually has an impact on the soil biome and becomes people’s environmental concern because of its residue toxicity in the ecological system. This issue has caused an organophosphorus pesticide contamination problem.

**Figure 1:**
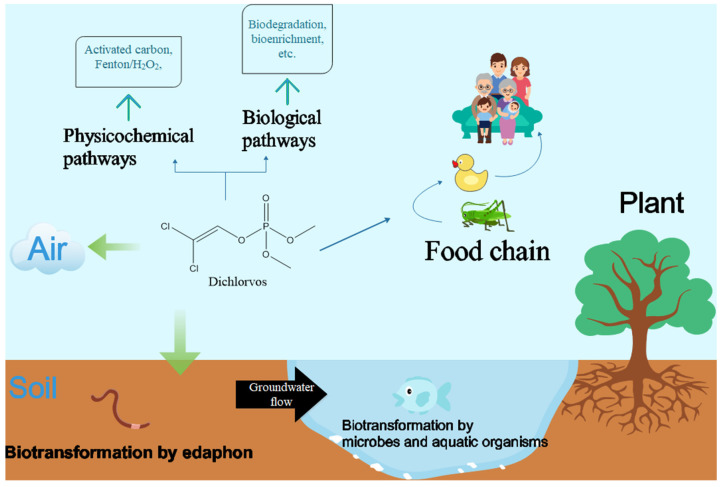
Contamination and removal of dichlorvos from natural environment.

The massive application of DDVP can affect non-target organisms profoundly through various kinds of pathways (Figure 1). Some reports have shown that exposure to DDVP in childhood is related to an increased risk of diabetes and may lead to the increasing risk of breast cancer in adulthood [6]. Scientific research has demonstrated certain effects of chronic exposure to DDVP on mouse. Those animals exposed to DDVP showed nigrostriatal neuron degeneration and remarkable behavioral impairment. Such animals have representative symptoms called catalepsy which is similar to those of Parkinson’s disease in humans [7]. However, the current situation shows that OPs, including DDVP, are still widely used in China, India, Brazil, and many other developing countries. Exposure is inevitable for people in those countries. Therefore, there is an extremely urgent need to deal with DDVP residues in the environment and to protect people from further physiological damage.

Many studies have tested the degradation of DDVP using different kinds of physicochemical methods, including gas phase surface discharge plasma, physical adsorption, hydrodynamic cavitation, and nanoparticles [8]. These approaches are not cost-effective and hard to apply to large contaminated areas. Therefore, microbial degradation of DDVP has become a powerful and attractive method to solve the exposure problem of this hazardous pesticide. Several microbes, including *Cunninghamell aelegans*, *Fusarium solani*, *Talaromyces atroroseus*, *Aspergillus oryzae*, *Ochrobactrum intermedium*, *Pseudomonas aeruginosa*, and *Penicillium* sp., have been isolated and play vital roles in DDVP degradation. It has been reported that the organic pollutants can be used by edaphon including soil bacteria and soil fungi as a sole carbon source [9,10,11,12,13]. These studies showed that microbial degradation seems to be a more environmentally friendly and convenient treatment method to reduce hazardous effects of toxic pollutants or contaminants [14,15,16,17,18,19]. In addition, there are a few studies on degrading enzymes with correlative genes in microbes. However, most of the studies have paid little attention to the mechanisms and degradation pathways of DDVP. This review summarizes different kinds of solutions to the DDVP contamination problem and describes the promising application prospect of microbial degradation. Moreover, it also discusses the mechanisms and degradation pathways of DDVP.

## 2. Toxicological Effects of DDVP

DDVP has the tendency to remain in solution due to its solubleness, with a limited tendency to absorb sediment. DDVP is subject to both abiotic and biological degradation in solution [20]. In addition, DDVP has the ability to regulate the neurotransmitter acetylcholine which leads to irreversible inhibition of acetylcholinesterase. Thus, it has harmful effect on nontarget invertebrates and vertebrates [21]. Based on the laboratorial research, DDVP can be hydrolyzed to dichloroacetaldehyde, dichloroethanol, dichloroacetic acid, dimethylphosphate, and dimethylphosphoric acid [22]. The process of DDVP degradation in moist soil is similar to that in aqueous solution. There are two main routes of exposure: inhalation and skin contact. People who are brought into contact with toxic waste containing DDVP or use domestic pesticides can potentially be exposed to them by inhalation. Due to its long half-life and current situation of usage, the toxic effects caused by DDVP residue should not be ignored. The toxicological effects of DDVP are presented in Table 1.

Toxic DDVP exposure in zebrafish was reported by Nguyen et al. [23], who illustrated various kinds of procedures in energy utilization and stress response in liver. Three concentrations of DDVP show that the effect on liver energy metabolism is rigorously controlled. Toxic exposure may lead to a certain amount of neuromuscular impairment in exposed zebrafish. Moreover, a study on tilapia demonstrated that acetylcholinesterase (AChE) suppression in brain and liver is caused by DDVP which exerts cholinergic action by blocking cholinesterase in the central and peripheral nervous system [24]. Tilapia lived under a sublethal concentration (0.5 mg/L) of DDVP, and all sizes of fish showed a significant inhibition of brain and liver AChE activities. AChE activity was regarded as an indication of the extent of pollution of the aquatic environment by organic chemicals and was correlated with water contamination.

Harmful impact on rats according to DDVP have also been investigated as representatives of damage to land mammals. Okamura et al. [25] reported a study in which Wistar rats were injected with four different dosages of DDVP dissolved in saline on their neck. Sperm motility is deteriorated by DDVP exposure at different doses, which means humans may suffer from testicular dysfunction. Another study focused on the biochemical and behavioral sequelae of chronic DDVP exposure in rats [26]. This study illustrated that all components of spontaneous locomotor activity in rats exposed to DDVP have reduced remarkably. DDVP administration also led to evident damage on rats’ muscle strength and coordination. According to a cellular-level study, exposure to DDVP may result in neuronal cell death in primary rats [27]. This study observed significant upregulation of pro-inflammatory molecules like nitric oxide, tumor necrosis factor alpha (TNF-*α*), and interleukin 1 beta (IL-1*β*) when microglia were treated with DDVP (10 μM). The study concluded that DDVP can induce microglial activation and then cause cell apoptosis. Another study described the influence of butyrylcholinesterase (BuChE) activity in rats with continued exposure to DDVP [28]. Different types of doses of DDVP (8.0 mg/kg of body weight) were given to both sexes of rats, with two-day intervals between administrations. This study clearly showed that exposure to DDVP significantly decreased the BuChE activity in both male and female rats.

There are several reports about the hazardous effects of DDVP on the human body indicating that a higher concentration of DDVP can cause death. A woman died a day after ingesting DDVP and an infant died after ingesting a cake-like bait that contained DDVP [29]. Although most of the studies showed little proof that exposure to DDVP is related to any cancer risk, Eroğlu et al. [2] and Koutros et al. [4] indicated the toxic effects of DDVP on human peripheral blood lymphocytes. As a result, DDVP-induced micronuclei decreased the mitotic and replication indices. This kind of genotoxic product causes chromosomal damage and cell death (decreased mitotic and replication indices). It has been classified by the United States Environmental Protection Agency as a toxic chemical in the Toxics Release Inventory (TRI) [30].

Some studies have illuminated the molecular mechanism of DDVP neurotoxicity. The dominating mechanism of action of DDVP is the inhibition of AChE, which causes an increase in the level of acetylcholine in the synaptic cleft and produces nicotine and muscarinic signs, which are also accompanied by symptoms of poisoning in the central nervous system [31]. However, a certain amount of acetylcholinesterase inhibition can be tolerant to nervous system without any toxic effects. In all kinds of mammals, toxic signs were discovered until acetylcholinesterase was inhibited by at least 20% [1].

## 3. Physicochemical Transformation of DDVP

Several physicochemical methods have been applied to control residual DDVP pollutant (Table 2). On the whole, these methods are efficient to a certain extent, but too expensive for developing countries that are suffering from DDVP contamination. Several researches have reported hazardous effects of DDVP exposure on aquatic animals, land mammals, and humans. Thus, removal of DDVP residue from contaminated environments is extremely urgent.

The main technique for solving pesticide pollutants is chemical degradation [43]. Other common solutions for solving pesticide pollutants include chlorination, hydrodynamic cavitation, active carbon, O_2_ plasma, metal catalysts, and H_2_O_2_ and O_3_ adsorption [14]. Bustos et al. [44] highlighted the urgent need and intricacy of photo-induced oxygen-mediated reactions of DDVP. DDVP is photoionized by electron transfer to dissolved oxygen, followed by superoxide radicals, and finally the HO yield. It might be the main mechanism of degradation taking place during photolysis. In addition, it has been investigated that the hydrodynamic cavitation reactor can be applied to degrade an aqueous solution of DDVP. As shown in another study, a chlorinated organophosphate compound can be effectively degraded using treatment strategies based on hydrodynamic cavitation in a large-scale operation [45]. According to this report, active carbon is an efficient substituent that absorbs DDVP residual, because powder-activated carbon shows excellent adsorption of aromatic compounds, including pesticides, herbicides, surface activators, natural pigments, and phenols [46].

Advanced oxidation processes (AOPs) containing various kinds of oxidants have been applied to remove hazardous pollutants from soil and water environments successfully. Bai et al. [47] noted that the O_2_ plasma treatment worked well in the DDVP remediation process, and the usefulness of degradation is mainly dependent on the related operating parameters and chemical structures of pesticides. Hydroxyl radicals have the ability to break the double bond in the DDVP molecule, and DDVP is further oxidized to 1,1-dichloro ethoxy dimethyl phosphate, 1,1,1-trichloro-2-hydroxyl-ethyl dimethyl phosphate, dimethyl phosphite, dimethyl phosphate, trimethyl phosphate, methyl phosphate, dichloro acetaldehyde, oxalic acid, CH_2_C_l2_, CHC_l3_ (parts of which are mineralized to phosphoric acid), CO_2_, H_2_O, and chloridion [48]. It took only 90 min to push the elimination ration up to 98% under acidic and saturated dissolved oxygen conditions [49]. Through the known products, the reaction mechanism of DDVP oxidized by H_2_O_2_ was discussed, and the conclusion was made that the main decontamination mechanism is radical chain reaction [50].

**Figure 2:**
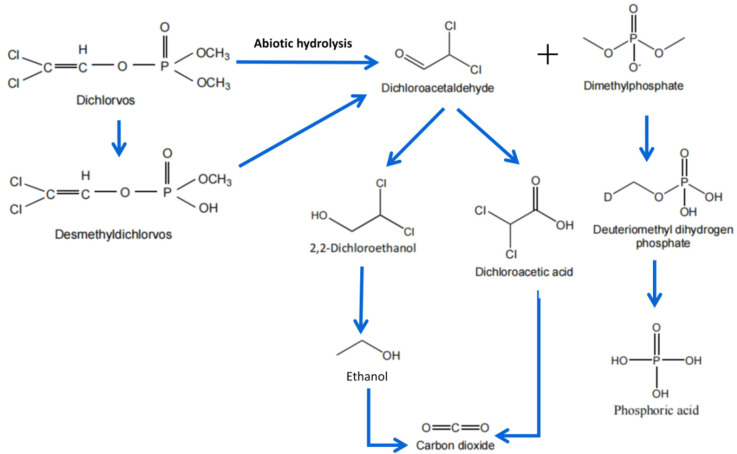
Proposed physical degradation pathways for dichlorvos decomposition in water and soil system.

Comparatively, ozone and hydroxyl radicals are vital for DDVP abatement. The abiotic hydrolysis degradation pathway is presented in Figure 2. However, we still need more detailed studies on aqueous solutions and lower concentrations of contaminants in order to properly assess the process performance.

Iron-modified ZSM-11 zeolites were applied as heterogeneous catalysts in the degradation process of DDVP water solutions. ZSM-11 zeolite matrices were synthesized by the hydrothermal method and iron was incorporated by the wet impregnation method in four concentrations. From this report, Fe/ZSM-11 with 6 wt% of incorporated iron showed the best catalytic behavior based on DDVP [51].

Removing DDVP pollutants from water is a real challenge due to the presence of the direct carbon-to-phosphorous covalent bond, which reveals its stability under chemical and thermal degradation. From recent studies, nanomaterials seem to be a possible solution for degradation. Mehrotra et al. [52] reported an efficient way for catalytic degradation of DDVP using protein-capped zero-valent iron nanoparticles, which removed the pesticide in 1 h. Moreover, the degradation mechanisms of DDVP during oxygen plasma treatment have been successfully detailed [53], so several active materials (high-energy electrons and free radicals) in oxygen plasma can thoroughly degrade DDVP within a short exposure time.

## 4. Microbial Degradation of DDVP

Microbial degradation is regarded as a cost-effective and promising method with a huge potential for the removal of pesticides, compared to physicochemical approaches [9,13,19]. Soil bacteria and fungi have been documented as being able to mineralize various organic pollutants as a sole carbon source [56,57,58,59,60]. Based on the existing research, the effective soil microorganisms for solving the DDVP residual have been isolated and studied (Table 3).

Five kinds of strains were selected and studied for the plant-fungi-spent mushroom compost (SMC) interaction, which has the potential to speed up the DDVP degradation rate [56]. According to this research, fungal strains identified as *Cunninghamella elegans*, *Fusarium solani*, *Talaromycesatro roseus*, *Aspergillus oryzae*, and *Penicillium* sp. were isolated from pesticide-polluted soil. Their rhizosphere interaction with plants (*Panicum maximum*) was shown in this study. The plant-fungi-SMC interaction synergistically sped up the DDVP degradation rate in a shorter time period, and an appreciable loss of DDVP of 72.23% and 82.70% degradation efficiency was observed in 30% and 40% of treatments, respectively, as compared to controls 1 and 2, with 62.20% and 62.33% degradation efficiency, respectively.

As a representative organophosphorus pesticide, DDVP has been applied in biodegradation studies. In a study by Jiang et al. [61], they found a bacterium that can degrade DDVP rapidly, *Ochrobactrum intermedium* DV-B31. This bacterium degraded 96.38% of DDVP samples in 8 days, which proved its potential for bioremediation. Interestingly, some bacteria, such as *Pseudomonas aeruginosa* and *Bacillus amyloliquefaciens* YP6, can degrade DDVP and other pesticides [62]. Nonetheless, the bioremediation ability of the bacterial cultures can be affected by different factors, including the type of inoculum and its density, pH, temperature, and toxic compounds present in the system [63].

Recently, there has been increasing interest in the biodegradation pathway of DDPV. A study of *Trichoderma atroviride* strain T23 presented two possible ways of degrading DDVP [60]. According to the results of this study, the first pathway is related to the breakage of the P-O bond. DDVP was converted to dimethyl phosphate (DMP) and dichloroacetaldehyde, and these intermediates can be rapidly tautomerized to dichloroacetic acid (DCAA) and dichloroethane (DCE). Some of the DCE is then transformed into trichloroethane (TCE) and the rest is dechlorinated into ethanol. Potentially, through the esterification of DCAA to ethyl dichloroacetate (EDCA), DMP is eventually converted into phosphate ions by strain T23. Moreover, the stochiometric amount of metabolites is lower than the consumption of DDVP, which leads to the second possible pathway. The second pathway involves the de-chlorination of DDVP to the isomers, (*Z*)-2-chlorovinyl dimethylphosphate and (*E*)-2-chlorovinyl dimethyl phosphate, while these isomers hardly undergo further de-chlorination to phosphoric acid trimethyl ester. Thus, they are unlikely the main by-products and are not easy to detect using normal techniques.

Sun et al. [64] noted that *Trichoderma atroviride* mutant AMT-28 is one of the most effective fungal bacteria and can completely remove DDVP pollution in 7 days. The DDVP removal is related to biomineralization process which attributed to fungal biodegradation. Parte et al. [65] demonstrated another biodegradation pathway in *Pseudomonas stutzeri* strain smk. This study elucidated the aerobic degradation pathway of DDVP: two dichlorination steps producing 2-chlorovinyl dimethyl phosphate and vinyl dimethyl phosphate. The vinyl dimethyl phosphate was then devinylated to produce dimethyl phosphate, which, upon two sequential demethylation steps was separated into 2-methyl moieties and a free phosphate to serve as the sole carbon and phosphate source to support growth. These various kinds of degradation pathways indicate that microbial degradation seems to be more adaptable to current agriculture and living conditions.

In some cases, a single type of a bacterial strain is not applicable due to the current degradation requirements. Based on this situation, Ning et al. [66] reported that degradation ability can be mutually promoted by a bacterial community. It seems that more kinds of bacteria have higher active constituents. A consortium of *Pseudomonas*, *Xanthomonas*, *Sphingomonas*, *Acidovorax*, *Agrobacterium*, and *Chryseobacterium* was reported, which extends the range of pesticide degradation by phyllosphere microbial communities and consequently provides a brand-new idea for the biodegradation of DDVP with pure microbial cultures from the plant phyllosphere.

## 5. Molecular Mechanism of DDVP Biodegradation

**Figure 3:**
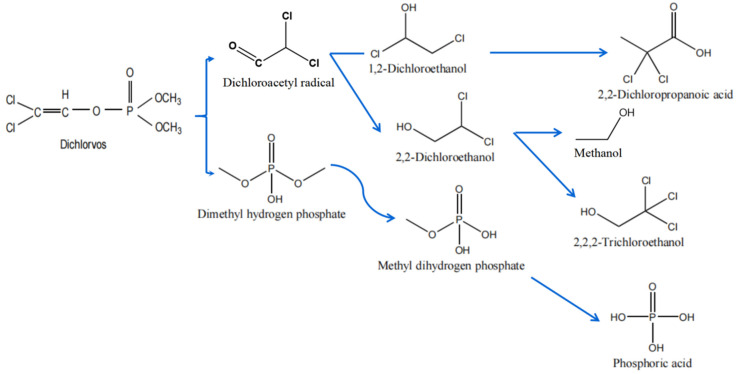
Proposed microbial degradation pathways of dichlorvos.

The proposed DDVP microbial degradation pathways are presented in Figure 3. The biodegradation mechanisms of many other organophosphorus pesticides have been deeply studied, especially those pesticides whose degradation genes and enzymes were cloned and purified [67,68,69,70,71]. According to previous research, most of the microbial degradation of DDVP is closely related to a functional gene that encodes for the enzymes, which is crucial in the degradation process [64].

Parte et al. [65] revealed the correlation between pesticide concentration and biodegradation ability. It seems that a lower concentration of DDVP supports bacterial growth, while higher concentration harms the bacteria. The reason may be that the cells and enzyme systems are hampered by increasing concentrations of pesticide, leading to lower biodegradation efficiency. Sun et al. [64] also found that the enzyme produced by TaPon1-like had a low *K_m_* for DDVP (0.23 mM) and a high *K*_cat_ (204.3 s^–1^). The enzyme was able to hydrolyze broad substrates with stable activity in a wide range of pH and temperature values. TaPon1-like hydrolase plays an important role in the first step of DDVP degradation by strain T23 and contributes to a comprehensive understanding of the mechanism of organophosphate pesticide degradation. The deletion of TaPon1-like weakened the efficiency of the DDVP degradation, but it did not abolish the hydrolysis ability, which indicates that TaPon1-like is one of the key enzymes of strain T23 that is responsible for the hydrolysis of the P-O bond in DDVP.

Moreover, a study demonstrated that AMT-28 could produce inducible intracellular-degrading enzyme of DDVP, causing immobilized cells to display ever-increasing DDVP degradation ability in reusability determinations. To thoroughly investigate the mechanism of DDVP bioremediation, research on the isolation and purification of inducible intracellular degrading enzyme are ongoing [72]. Although the degradation mechanism has not been clearly explained, some kinds of fungi can produce novel OPs degrading enzyme [73,74,75].

The elimination of DDVP from saline solutions has been attributed to its ability to penetrate into the cytoplasm via a principle, called “organic-osmolyte.” Moreover, the PON1 gene, which exists widely in mammals, has been found to have a powerful influence on the detoxification of organophosphate compounds. This led to the result that PON1 has the ability to prevent oxidative damage to tissues, which seems to be reasonable [76]. Therefore, PON1 may prevent tissue damage due to organophosphate toxicity, especially in the central nervous system [76]. This study has shown that PON1 could effectively reduce the blood concentration and decrease the peak concentration of DDVP, and lessen the amount that enters the blood. This study also compared the hydrolytic effect of PON1 with atropine + PAM, the most widely used clinical therapy. It showed that atropine + PAM did not affect the metabolism of DDVP, which was consistent with a recent research [76]. Co-treatment does not alter the impact of PON1 on DDVP concentration, which implies that there is no interaction between PON1 and atropine + PAM-CI; therefore, it is supposed that co-treatment may be feasible in the clinical treatment of human organophosphate-related toxicity [77,78,79].

Based on these studies, it seems that using microorganisms merely is not the most effective method. Unfortunately, current knowledge of the DDVP biodegradation mechanism is still very limited; more research should be focused on identifying the novel genes and enzymes to explore the degradation pathway.

## 6. Conclusions and Future Perspectives

Different physicochemical methods have been developed for the removal of DDVP from contaminated environments, and microbial degradation is regarded as a promising method to solve several harmful residuals caused by DDVP. The biodegradation mechanism of many OPs has been studied deeply, especially for the methyl parathion, whose degradation genes and enzymes were cloned and purified. There is a need to select more useful strains, since only a few bacteria have been studied thoroughly in relation to the functional enzymes and genes. Moreover, the large number of different DDVP biotransformation metabolites should be detected to avoid secondary pollution.

Under the current situation of DDVP usage distribution, developing countries are more liable to suffer from exposure toxicity, but they are not allowed to use several physicochemical methods to solve the problem due to their economic capability. As a result, there is an urgent need for further study of biodegradation, in order to provide cloned strains to reduce the threat of DDVP exposure at lower cost. In the future, advanced scientific technologies such as gene editing and DNA isotope probes could be used to search for and evaluate more adaptable microorganisms for pesticide degradation. Moreover, next-generation sequencing analysis of the complete genome could explore the bioremediation potential of indigenous microbial strains in detail. The potential strains can be applied for large-scale treatment of DDVP and other pesticides.

## Figures and Tables

**Table 1:** Toxicological effects of dichlorvos in humans and animals.

No.	Study Sample/ Sample Sources	Concentration/ Volume of Dichlorvos	Specific Statement	Reference
1	Zebrafish	6, 19, 32 mg/L	Neuromuscular impairment arise from dichlorvos	[23]
2	Tilapia	0.5 mg/L	Significant inhibition of brain and liver acetylcholinesterase (AChE) activity	[24]
3	*Drosophila*	775 mg/kg	Mortality increased with increased dichlorvos dose	[32]
4	Larval butterflies	5–994 mg/L	Dichlorvos did not appear to affect life cycle of surviving caterpillars	[33]
5	*Tor putitora*	12.964 mg/L	Exposure to dichlorvos induced significant drop in oxygen consumption	[34]
6	Loach	4.56, 5.76, 7.12, 8.96, 11.20 μg/L	Decreased glutamic-pyruvic transaminase and glutamic-oxalacetic transaminase activity of liver	[35]
7	Chicks	6.51 mg/kg	Dichlorvos significantly reduced plasma and brain cholinesterase activity	[36]
8	Cyanobacteria	261.16 μmol/L	Significantlydecreased chlorophyll content	[37]
9	Wistar rats	0, 1, 2, 4 mg/kg	Decreased sperm motility	[25]
10	Rats	6 mg/kg	Severe toxic manifestations in motor and memory functions	[26]
11	Primary rats	10 μmol/L	Microglial activation and ultimately apoptotic cell death	[27]
12	Rats	8 mg/kg	Decreased butyrylcholinesterase (BuChE) activity	[28]
13	Rats	1.8, 9 mg/kg	Acute exposure to dichlorvos led to nitro-oxidative stress in the brain	[38]
14	Wister rats	1.8, 100 mg/kg	Decreased respiratory rate	[39]
15	Mice	40 mg/kg	Exposure to dichlorvos led to neuronal damage	[40]
16	Albino rats	50 mL dichlorvos/50 mL distilled water	Lungs and liver revealed moderate lymphocytic infiltration and hepatocytic steatosis after gradually exposed to dichlorvos	[41]
17	Human	Unknown	A woman died a day after ingesting dichlorvos	[29]
18	Human	Unknown	Dichlorvos known to inhibit plasma, erythrocyte, and brain AChE activity	[42]
19	Human	Unknown	An infant died after ingesting cake-like bait containing dichlorvos	[29]
20	Cells	5, 10, 20, 40, 80, 100 mg/L	Toxic nuclear effects in human peripheral blood lymphocytes	[2]
21	Cells	50–500 μmol/L	Cell death increasing accompanied by mitochondrial membrane potential decrease	[21]

**Table 2:** Physical and chemical methods used to degrade dichlorvos from environments.

No.	Study Sample/ Sample Sources	Physicochemical Method Used	Medium	Specific Statement	Reference
1	Sunlight/UV	Photocatalysis	Water	pH 3 conditions increased dichlorvos photodegradation up to 32%, with degradation rate constant of 0.064 h^−1^	[44]
2	Hydrodynamic cavitation reactor/Fenton	Advanced oxidation processes (AOPs)	Water	91.5% dichlorvos was degraded in 1 h	[45]
3	Activated carbon	Adsorption	Water	Average removal rate of dichlorvos was 95.1%	[46]
4	O_2_ plasma	AOPs	Air	Most of the dichlorvos was removed in 120 s	[47]
5	Fe ZSM-11	Photocatalysis	Water	Dichlorvos was degraded in 120 min (6% Fe ZSM-11)	[51]
6	Zero valent iron nanoparticles	Photocatalysis	Water	Pesticide was removed in 1 h	[52]
7	Fenton/H_2_O_2_	AOPs	Water	In acidic and saturated dissolved oxygen conditions, it took nearly 90 min to push degradation ratio up to 98%	[49]
8	H_2_O_2_	AOPs	Air	80.7% of dichlorvos vapor was decontaminated by 110–130 mg/m^3^ of H_2_O_2_ aerosol in 60 min	[48]
9	O_3_	AOPs	Water	Ozone plays an important role in dichlorvos degradation	[50]
10	Dielectric barrier discharge (DBD) plasma	Free radicals	Water	At lower initial concentration, the disappearance rate of dichlorvos followed first-order rate law; at higher initial concentration, the disappearance rate of dichlorvos shifted to zero-order rate law	[54]
11	Fresh frozen plasma	AOPs	Air	Dichlorvos half-life is 17.9 min	[55]

**Table 3:** Microbial degradation of dichlorvos.

No.	Strain or Community	Sample Sources	Detected Metabolites	Comments	Reference
1	*Cunninghamella elegans*	Surroundings of sewage disposing outlet from agro-pesticide manufacturing in Owo, Nigeria	*O*,*O*-dimethyl phosphonic ester, desmethyl dichlorvos, also known as 2,2-dichlorovinyl *O*-methylphosphate, and *O*,*O*,*O*-trimethyl phosphoric ester, also known as dichlorvos (2,2-dichlorovinyl-*O*,*O*-dimethyl phosphate)	*Cunninghamella elegans* strain was the most dominant fungal strain in pesticide-polluted soil samples with 37 appearances in 50 samples (74% incidence), *Talaromycesatro* roseus had 33 appearances (66% incidence), *Aspergillus oryzae* had 32 appearances (64% incidence), *Fusarium solani* and *Penicillium* sp. both had 26 appearances (52% incidence).	[56]
2	*Fusarium solani*				
3	*Talaromycesatro roseus*				
4	*Aspergillus oryzae*				
5	*Penicillium* sp.				
6	*Ochrobactrum intermedium* DV-B31	Farmland annually sprayed with organophosphorus pesticides	No data	96.38% dichlorvos was degraded by DV-B31 in 8 days	[61]
7	*Pseudomonas aeruginosa*	Agricultural field inPunjab, India	No data	90% of dichlorvos was degraded in around 20 days	[58]
8	*Bacillus amyloliquefaciens* YP6	Phosphate mine in Guizhou Province, China	No data	53% of dichlorvos was degraded in 1 h	[62]
9	*Trichoderma atroviride* T23	No data	Dichloroethane and trichloroethylene	300 μg/mL dichlorvos was degraded in 120 h	[60]
10	*Trichoderma atroviride* mutant AMT-28	Vegetable field in Shenyang, China	No data	Dichlorvos was completely removed when treated with mycelia of AMT-28 for 7 d	[64]
11	Consortium of *Pseudomonas*, *Xanthomonas*, *Sphingomonas*, *Acidovorax*, *Agrobacterium*, and *Chryseobacterium*	Greenhouse within Xisanqi Ecological Garden, Beijing, China	No data	Dichlorvos degradation efficiency of these bacteria was 11.5%,70.0%, 78.7%, 52.6%, 66.4%, and 25.2%, respectively	[66]
12	*Pseudomonas stutzeri* smk	India	Free methyl and phosphate	80% of dichlorvos was degraded on 7th day of incubation	[65]

## Abbreviations

DDVP	*O*,*O*-Dimethyl *O*-(2,2-dichlorovinyl)phosphate
OPs	Organophosphorus pesticides
WHO	World Health Organization
AChE	Acetylcholinesterase
BuCh	Butyrylcholinesterase
TRI	Toxics Release Inventory
AOPs	Advanced oxidation processes
SMC	Spent mushroom compost
DMP	Dimethyl phosphate
DCAA	Dichloroacetic acid
DCE	Dichloroethane
TCE	Trichloroethane
EDCA	Ethyl dichloroacetate
